# Transplantation Site Affects the Outcomes of Adipose-Derived Stem Cell-Based Therapy for Retinal Degeneration

**DOI:** 10.1155/2020/9625798

**Published:** 2020-01-06

**Authors:** Chengyu Hu, Huanzhi La, Xuancheng Wei, Yue Zhou, Qingjian Ou, Zhiyang Chen, Xiaoman Zhu, Jing-Ying Xu, Caixia Jin, Furong Gao, Juan Wang, Jingfa Zhang, Jieping Zhang, Lixia Lu, Guo-Tong Xu, Haibin Tian

**Affiliations:** ^1^Department of Ophthalmology of Shanghai Tenth People's Hospital, Tongji Eye Institute, Tongji University School of Medicine, Shanghai, China; ^2^Laboratory of Clinical Visual Science, Department of Regenerative Medicine, Stem Cell Research Center, Tongji University School of Medicine, Shanghai, China; ^3^Department of Physiology and Pharmacology, TUSM, Shanghai, China; ^4^Translational Medical Center for Stem Cell Therapy, Shanghai East Hospital, Tongji University School of Medicine, Shanghai, China; ^5^The Collaborative Innovation Center for Brain Science, Tongji University, Shanghai, China

## Abstract

Adipose-derived stem cells (ASCs) have shown a strong protective effect on retinal degenerative diseases (RDD) after being transplanted into the subretinal space in an animal model. Recently, several clinical trials have been conducted to treat RDD with intravitreal transplantation of stem cells, including ASCs. However, the outcomes of the clinical trials were not satisfactory. To investigate if the transplantation site alters the outcome of stem cell-based therapy for RDD, we isolated rat ASCs (rASCs) and labeled them with green fluorescent protein. Autologous rASCs were grafted into the vitreous chamber or subretinal space in a rat RDD model induced by sodium iodate (SI). The electric response was recorded by ERG. The anatomic structure of the retina was observed in cryosections of rat eyes at posttransplantation weeks 1, 2, and 4. Neural retina apoptosis and epiretinal membrane- (ERM-) like structure formation were investigated by immunostaining. The intravitreal transplantation of rASCs resulted in an extinguished electric response, although the rosette formation and apoptosis of neural retina were reduced. However, the rASCs that grafted in the subretinal space protected the retina from the damage caused by SI, including a partial recovering of the electric response and a reduction in rosette formation. Intravitreally grafted rASCs formed a membrane, resulting in retina folding at the injection site. Müller cells, retinal pigment epithelial cells, and microglial cells migrated from the retina to the rASC-formed membrane and subsequently formed an ERM-like structure. Furthermore, vitreous fluid promoted rASC migration, and rASC-conditioned medium enhanced Müller cell migration as indicated by in vitro studies. These data suggested that the vitreous chamber is not a good transplantation site for ASC-based therapy for RDD and that a deliberate decision should be made before transplantation of stem cells into the vitreous chamber to treat RDD in clinical trials.

## 1. Introduction

Retinal degenerative diseases (RDD), including age-related macular degeneration (AMD) and retinitis pigmentosa (RP), are one of the leading causes of blindness worldwide [1, 2]. The progressive and eventual dysfunction and death of retinal pigment epithelial (RPE) cells and photoreceptors are the main etiology of RDD [1, 2]. For a long time, it was an incurable disease.

Recently, stem cell-based therapy shows great therapeutic potential for treating RDD [3]. Adipose-derived stem cells (ASCs) have a high proliferation rate and self-renewal capacity, and they can differentiate into various cell types, including bone, cartilage, and adipose cells [4]. Under proper conditions, ASCs can even transdifferentiate into cells from other germ layers, such as neural cells and retinal cells [5–7]. Thus, ASCs may be able to replace damaged cells after transplantation in vivo. Furthermore, ASCs can secrete many cytokines and growth factors directly or in exosomes to benefit the regeneration of damaged tissues [8, 9]. These properties make ASCs the proper candidate for treatment of RDD. Several published papers have reported that ASCs delay the retinal degeneration process after subretinal space transplantation in sodium iodate- (SI-) induced animal RDD models [10, 11]. Our group demonstrated that human ASCs delay the retinal degeneration process after subretinal space transplantation in RCS rats and that ASC paracrine function, such as production of vascular endothelial growth factor (VEGF), mainly contributes to their therapeutic functions [12].

In addition to ASCs, other kinds of mesenchymal stem cell-based therapies for RDD have also shown promising outcomes in animal RDD models [13–15], prompting scientists and doctors to conduct clinical trials. The outcomes of several clinical trials have shown that some patients who received intravitreal transplantation of human ASCs have dense vitreous hemorrhage in the eyes, retinal detachment with severe proliferative vitreoretinopathy (PVR), and secondary epiretinal membrane (ERM) formation [16, 17]. These complications are sometimes associated with the surgery. However, more studies showed that intravitreal transplantation of other kinds of stem cells also resulted in ERM formation [18–20], which suggest that not only surgery process but also transplantation sites may affect therapeutic outcomes. When stem cells are used to treat RDD, cells can be transplanted into the subretinal space or the vitreous chamber. Most published papers demonstrated that when stem cells, including ASCs, were grafted into the subretinal space, grafted cells stayed in the subretinal space and showed protective functions [12–15]. However, previous clinical trials have preferred the vitreous chamber as the transplantation site (http://www.clinicaltrials.gov, NCT02016508, NCT01518127, NCT03772938, NCT01920867, NCT03011541, NCT02024269, NCT0106856, NCT01560715, NCT01531348, and NCT02280135). Thus, if the subretinal space, rather than the vitreous chamber, is the proper transplantation site for stem cell-based therapy of RDD, patients receiving vitreous chamber transplantation of stem cells will be at a clear disadvantage. Therefore, it is necessary to clarify the optimal transplantation site for treatment of RDD in clinical trials.

There are two types of ERM, namely, idiopathic and secondary types [21, 22]. The etiologies of idiopathic type involve glial cell migration from ruptured internal limiting membrane and proliferation and spreading across the retinal surface. The secondary ERM is the complication of eye diseases, such as retinal detachment, diabetic retinopathy, and uveitis; especially, retinal detachment has a certain incidence during stem cell transplantation process [23]. In addition to glial cells, RPE cells migrate from a retinal tear and proliferate and contribute to the secondary ERM formation [24, 25]. ERM attaches to the retina and contracts and distorts the retina [26]. Previous studies have shown that ERM formed in patients received intravitreal transplantation of bone marrow-derived stem cells and ASCs [17, 19]. However, the ERM formation process, ERM cellular components, and the contribution of stem cells to ERM formation in these patients are not clear.

In this study, a rat RDD model was established by intravenous infusion of SI, and rat ASCs (rASCs) were autografted into the vitreous chamber or subretinal space to clarify whether the transplantation site affects the outcome of ASC treatment of RDD. Epiretinal membrane formation was also investigated to demonstrate a potential secondary complication of ASC treatment of RDD, thus providing critical information for future clinical trials.

## 2. Materials and Methods

### 2.1. Animal

The 6-week-old Sprague-Dawley (SD) rats (Animal Center of Tongji University) were used in this study. Animal experiment was conducted in accordance with the ARVO (The Association for Research in Vision and Ophthalmology) Statement for the Use of Animals in Ophthalmic and Vision Research. Animal studies were carried out at the Animal Facilities of the Tongji University. Animal protocols were approved by the Institutional Animal Care and Use Committee of Tongji University, Shanghai.

### 2.2. Preparation of rASCs

Rat subcutaneous adipose tissue was obtained through surgery as previously described with modifications [27]. Briefly, rats were anesthetized by 2% sodium pentobarbital, and the skin was sterilized by 70% ethanol and opened by a pair of scissors. The subcutaneous adipose tissues were harvested and extensively washed with phosphate-buffered saline (PBS). Tissues were digested with 10 volume of collagenase type I (5 mg/mL in low glucose (1 g/L) DMEM, Invitrogen, Carlsbad, CA) for 60 min at 37°C with intermittent shaking. After being neutralized with complete ASC medium (low glucose (1 g/L) DMEM, 10% of FBS, 50 U/mL penicillin, 50 mg/mL streptomycin (all from Invitrogen)), digested tissues were centrifuged at 300×g for 5 min, and the floating adipocytes and blood cells were separated from the stromal fraction by centrifugation. The cell pellet was resuspended with a complete rASC medium and filtered through a 70 *μ*m net. The obtained rASCs were cultured at 37°C, 5% CO_2_.

### 2.3. Müller Cell Culture

The rat Müller cell (rMC-1 cell) was kindly supplied by Vijay Sarthy (Northwestern University, Evanston, IL). The cells were maintained in high glucose (4.5 g/L) DMEM containing 10% FBS and 1% penicillin-streptomycin at 37°C with 5% CO_2_ in a humidified incubator.

### 2.4. Induction of Adipogenesis, Osteogenesis, and Chondrogenesis

rASCs were induced to differentiate into adipocytes, osteoblasts, and chondrocytes. For adipogenesis, the cells were cultured in adipogenic induction medium (DMEM supplemented with 10% FBS, 10^−7^ M dexamethasone (Sigma, St. Louis, MO), 100 *μ*Μ indomethacin (Sigma), 100 *μ*M 3-isobutyl-1-methyl-xanthine (Sigma), and 10 mg/L insulin (Invitrogen)), and the medium was refreshed every 2 days. Two weeks later, the cells were fixed with 4% paraformaldehyde (PFA) and stained with Oil red O (Sigma). For osteogenesis, the cells were maintained in osteogenic induction medium (DMEM supplemented with 10% FBS, 10 mM *β*-glycerol phosphate (Sigma, CA), 50 *μ*M L-ascorbate-2-phosphate (Sigma), and 5 ng/mL recombinant human bone morphogenetic protein-2 (HumanZyme, Chicago, IL)) and the medium was changed with fresh one every 2 days. One week later, the cells were examined for alkaline phosphatase (AKP) activity by vector blue alkaline phosphatase substrate kit III (Vector, Burlingame, CA). For chondrogenesis, the cells were cultured in chondrogenic induction medium which includes DMEM supplemented with 10% FBS, 10 ng/mL TGF*β*1 (HumanZyme, Chicago, IL), 0.1 mol/L dexamethasone (Invitrogen), 50 mg/L L-ascorbate-2-phosphate (Sigma), and 50 g/L ITS (Invitrogen). The cells were cultured for two weeks and fixed in 4% PFA and stained with toluidine blue sodium borate (Sigma).

### 2.5. Flow Cytometry

rASCs were detached and dissociated to single cells with 0.25% trypsin/0.53 mM EDTA buffer, then resuspended in PBS containing 1% bovine serum albumin (BSA) and FITC-labeled antibodies against CD29, CD90, CD11b, and CD45, (BD Biosciences, San Jose, CA), respectively, for 30 min on ice. FITC-mouse IgG1 was used as isotype control. The cells were washed with PBS twice and analyzed on a FACScan instrument using CellQuest software (BD Biosciences).

### 2.6. Lentivirus Packaging and Infection

HEK293T cells were cultured to 70% confluence in 10 cm dish and then transfected with 10 *μ*g FG12 plasmid carrying green fluorescent protein (GFP) gene and 5 *μ*g of each VSVG, REV, and PRRE packaging plasmids by liposome 2000 (Invitrogen). After 12 h, the medium was refreshed completely. After 60 h, the supernatants were collected and filtered through 0.45 *μ*m pore-size filters (Millipore, Boston, MA). For labeling rASCs, 5 × 10^5^ cells were seeded in a 10 cm culture dish for a day, and then, the medium was replaced with fresh medium plus virus-containing supernatant and 8 *μ*g/mL polybrene (Sigma), followed by 12-hour incubation. GFP-labeled rASCs were sorted by flow cytometry and used for autotransplantation.

### 2.7. Autologous rASC Transplantation

The SD rats that grew up to 3 months old were used to establish the RDD model and received autologous rASC transplantation as previously reported with modifications [12]. Considering that the retinal cells start to undergo apoptosis as early as one day after injection of SI [28], we transplanted rASCs at day 1 post injection of SI to inhibit the apoptosis of retinal cells. In addition, SI was metabolized within dozens of minutes in vivo [29, 30]; thus, the transplanted cells will not be damaged by SI at this time point. Briefly, rats were intravenously injected with SI (50 mg/kg, Sigma) to induce RDD model, and 24 h later, rats were anesthetized by 2% sodium pentobarbital. A channel was created by inserting a 30-gauge needle, behind the limbus, into the vitreous chamber. For the intravitreal transplantation, a 33-gauge needle was inserted into the vitreous chamber and 3 microliters of the autologous GFP-labeled rASC suspensions (1 × 10^5^ cells/*μ*L) was injected into the vitreous chamber near the center of the retina. For the subretinal transplantation, a 33-gauge needle was inserted into the subretinal space of the central retina and 3 microliters of the autologous rASC suspensions (1 × 10^5^ cells/*μ*L) was injected. The eyes that received a sham injection of PBS were used as control.

### 2.8. Electroretinogram (ERG) Examination

After the cell transplantation, ERG b-wave amplitude was measured weekly up to 4 weeks posttransplantation, with AVES-2000 electrophysiological apparatus (Kanghuaruiming S&T, Chongqing, China) as described previously [13, 14]. An intensity of 6.325*e*-2 cd^∗^s/m was applied, which allowed the recording of the response of photoreceptors to light stimulation.

### 2.9. Preparation of Retina Cryosections

The rats were killed with an overdose of sodium pentobarbital at weeks 1, 2, and 4 posttransplantation. The eyeballs were removed immediately and fixed in 4% PFA. The embedded tissues were sectioned (10 *μ*m thickness) along the vertical meridian of the eyeball from nasal to temporal side, and the cryosections were used for analysis. To assess the distortion of the retina, the nuclei in the sectioned tissue were stained with DAPI.

### 2.10. Terminal Deoxynucleotidyl Transferase-Mediated Deoxyuridine Triphosphate Nick End Labeling (TUNEL) Assay

Eye samples were collected at weeks 1, 2, and 4 posttransplantation and fixed in 4% PFA, and then, cryosectioned samples were prepared. TUNEL assay was conducted with an In Situ Cell Death Detection Kit (Roche, Diagnostics, Switzerland), according to the manufacturer's instructions. Nuclei were counterstained with DAPI. The apoptosis of the retinal outer nuclear layer (ONL), inner nuclear layer (INL), and ganglion cell layer (GCL) was assessed based on the counts of TUNEL+ cells per field. The percent of apoptotic cells was calculated and presented as a spider graph [31].

### 2.11. Immunostaining

For immunofluorescence analysis, cryosections of the eyes were permeabilized with 0.25% Triton X-100 (Sigma) for 5 min, washed with PBS, and then blocked with 2% BSA (Sigma) in PBS. The sections were incubated with the primary antibodies against SLC1A3, Ezrin, Iba-1, and *α*-SMA (Abcam, Cambridge, UK) both at 1 : 500 overnight at 4°C. They were then washed three times with PBS, followed by incubation with the fluorescent secondary antibodies (1 : 1000, Invitrogen) overnight. DAPI was used to indicate the nucleus. The samples were then examined by a fluorescence microscope (Olympus IX73, Tokyo, Japan).

### 2.12. Preparation of Vitreous Fluid

The eyes were obtained from 3-month-old rats and wiped with alcohol cotton; after washed with PBS, corneal and lens were removed, and vitreous fluid was aspirated by a micropipette. Vitreous fluid was diluted with low glucose (1 g/L) DMEM (1 : 10) and filtered by a 0.22 *μ*m microporous membrane.

### 2.13. Scratch Analysis

rASCs grew to 100% confluence in a 48-well culture plate and were starved in low glucose (1 g/L) DMEM containing 0.5% FBS for 12 h. The cell monolayer was scraped in a straight line with a p1000 pipet tip to create a “scratch.” The monolayer is washed with DMEM to remove cell debris and cultured in DMEM containing 0.5% FBS without or with 10% vitreous fluid for 24 h. Cells were fixed in 4% PFA and stained with Giemsa. The scratch area was measured using a microscope and ImageJ 1.8.0 software (National Institutes of Health). Results were expressed as percentage of reduced scratch area ((The scratch area measured immediately after scraped - The scratch area measured after scraped for 24 h)/The scratch area measured immediately after scraped × 100%) as described previously [32].

### 2.14. Preparation of rASC-Conditioned Medium

rASCs grew to 80% confluence and were cultured in high glucose (4.5 g/L) DMEM for 24 h, and the supernatant was collected and was used as rASC-conditioned medium.

### 2.15. Müller Cell Migration

Müller cells grew to 80% confluence and were starved in high glucose (4.5 g/L) DMEM containing 0.5% FBS. Cells were detached by trypsin/EDTA, resuspended in high glucose (4.5 g/L) DMEM and added into the upper chamber of a 24-transwell plate (8 *μ*m pore, BD Biosciences) at 1 × 10^5^ cells/well. DMEM or ASC-conditioned medium was added to the lower chamber. The cells were allowed to migrate for 4 h at 37°C in 5% CO_2_. Migrated cells, which were attached to the undersides of membranes, were stained with Giemsa. The images were captured by a microscope. Results were expressed as the number of migrated cells per field.

### 2.16. Statistical Analysis

All experiments were repeated a minimum of three times. The data were analyzed by GraphPad Prism 6 software. All values were expressed as the mean ± SD and subjected to one-way ANOVA followed by Tukey's test. A *P* value less than 0.05 was considered statistically significant.

## 3. Results

### 3.1. Identification of rASCs

**Figure 1:**
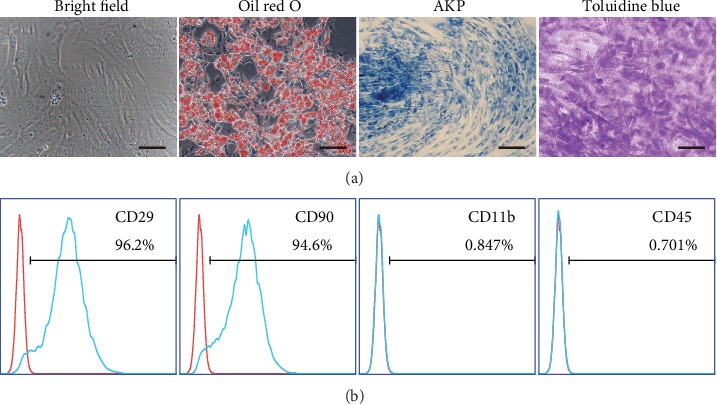
Characterization of rASCs. (a) Morphology (bright field), adipogenesis (Oil red O), osteogenesis (AKP), and chondrogenesis (toluidine blue) of rASCs (scale bar = 50 *μ*m). (b) Flow cytometry analysis of rASCs.

rASCs were isolated from subcutaneous adipose tissue and showed fibroblast-like cell morphology. To confirm potential differentiation capacities of rASCs, we cultured rASCs under adipogenic, osteogenic, and chondrogenic differentiation conditions, and rASCs differentiated into adipocytes (Oil red O staining), osteoblasts (AKP staining), and chondrocytes (toluidine blue staining) (Figure 1(a)). Flow cytometry analysis demonstrated that ASCs were positive for CD29 and CD90 and negative for CD45 and CD11b (Figure 1(b)). These results demonstrated that the isolated cells were mesenchymal stem cells.

### 3.2. rASCs Do Not Recover Vision Loss When Transplanted into Vitreous Chambers of SI-Induced RDD Rats

**Figure 2:**
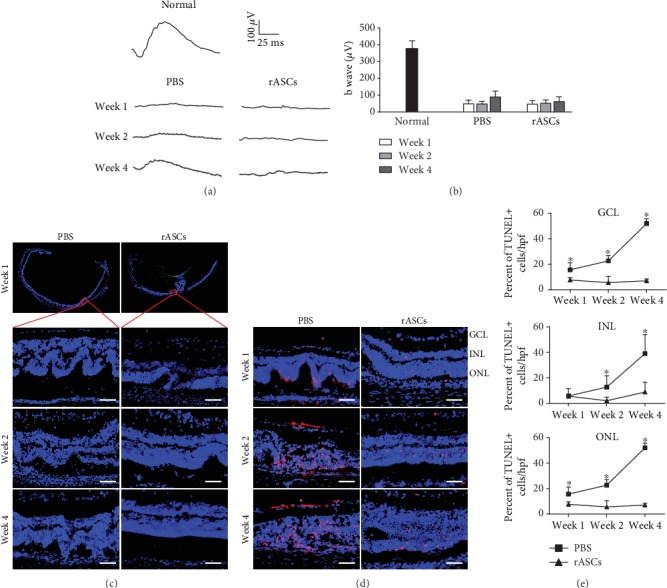
Protective effects of rASCs on retina in SI-induced rat RDD model. 3 × 10^5^ rASCs were transplanted into the vitreous chamber. (a) Representative ERG waveforms recorded at different time points (the calibration indicates 100 *μ*V vertically and 25 ms horizontally). (b) Quantitative analysis of ERG b-wave amplitude (*n* = 10). (c) Representative DAPI-stained micrographs of retinal samples (scale bar = 50 *μ*m). (d) Representative micrographs of retinal cryosections stained with TUNEL (scale bar = 50 *μ*m). (e) Statistical analysis of the percentage of the apoptotic cells in GCL, INL, and ONL (*n* = 12). Results are expressed as mean ± SEM; ^∗^*P* < 0.05 compared with the rASC group. GCL: ganglion cell layer; INL: inner nuclear layer; ONL: outer nuclear layer.

To evaluate the effect of rASCs grafted into the vitreous chamber on SI-induced damage to the retina, we established a rat RDD model by intravenous infusion of SI, and rASCs were autografted into the vitreous chamber. ERG was measured to demonstrate rat visual function up to 4 weeks posttransplantation of rASCs. In the control group, PBS was injected into the vitreous chamber. The b-wave is correlated with the number of functional photoreceptors (PRs) and was used to evaluate the response of PR to light stimulation. In the PBS group, the b-wave significantly decreased to baseline after SI infusion for one week and was maintained at lower level in the following weeks (Figures 2(a) and 2(b)). ASCs can produce nutritional factors and have retinal protective functions [12]. However, the b-wave amplitude in the rASC treatment groups did not increase and was similar to that of the PBS groups (Figures 2(a) and 2(b)). These results indicated that ASC treatment does not improve the visual function of rats in comparison with the PBS group.

Previous work has demonstrated that subretinal space transplantation of ASCs delays RDD progress via a paracrine function in an animal model [12]. This prompted us to investigate whether intravitreal transplantation of rASCs reduces the damage induced by SI to the anatomic structure of the retina even though rASCs do not benefit the visual electric response. We first prepared cryosections of the retina to investigate if rASCs protect the anatomic structure of the retina from SI-induced damage. The neural retina was distorted by SI infusion and formed many rosette structures in the PSB group. GFP-labeled rASCs were observed in the vitreous chamber, and part of the neural retina formed a fold at the rASC injection site in the vitreous chamber. However, rASC treatment reduced rosette structure formation in the nonfolded part of the neural retina, which maintained normal layers within the observed time point (Figure 2(c)). We further examined the apoptosis of the retinal neurons in the nonfolded part of neural retinas with or without rASC transplantation. As shown in Figure 2(d), TUNEL-positive apoptotic cells were detected in the GCL, INL, and ONL of the retinas in the PBS groups, and the number of apoptotic cells was increased from week 1 to week 4. Such neuron apoptosis is well explained by the disappearance of electric response in SI-induced RDD rats. In contrast to the increased apoptotic neuronal cells in the PBS groups, intravitreal transplantation of rASCs effectively reduced the number and percentage of apoptotic cells in the GCL, INL, and ONL (Figures 2(d) and 2(e), *P* < 0.05). These data collectively demonstrated that rASCs autografted into the vitreous chamber reduced retina structure distortion and inhibited apoptosis, but they induced a retinal fold and did not improve the electric response in SI-induced rats. Thus, the vitreous chamber may not be a suitable transplantation site for ASC treatment of RDD.

**Figure 3:**
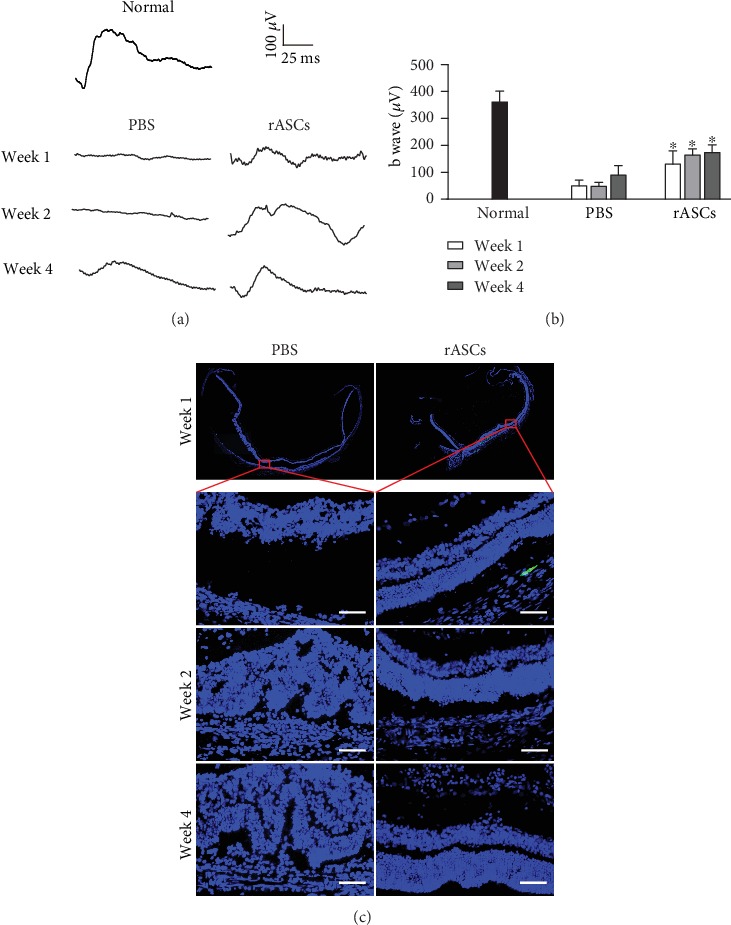
Protective effects of rASCs on the retina in SI-induced RDD rat. 3 × 10^5^ rASCs were transplanted into the subretinal space. (a) Representative ERG waveforms recorded at different time points (the calibration indicates 100 *μ*V vertically and 25 ms horizontally). (b) Quantitative analysis of ERG b-wave amplitude (*n* = 10). (c) Representative DAPI-stained micrographs of retinal samples (scale bar = 50 *μ*m). Results are expressed as mean ± SEM; ^∗^*P* < 0.05 compared with the PBS group.

We next investigated if the subretinal space is the proper transplantation site for stem cell-based therapy for RDD. rASCs were autografted into the subretinal space in SI-induced RDD rats. b-wave amplitudes were markedly increased in the rASC treatment group compared with the PBS group (Figures 3(a) and 3(b)), and retinas with transplanted rASCs maintained better anatomic structures compared with the PBS group (Figure 3(c)). These results demonstrated that the subretinal space, rather than the vitreous chamber, is the suitable transplantation site for ASC treatment of RDD.

### 3.3. rASCs Form an ERM-Like Structure in the Vitreous Chamber

**Figure 4:**
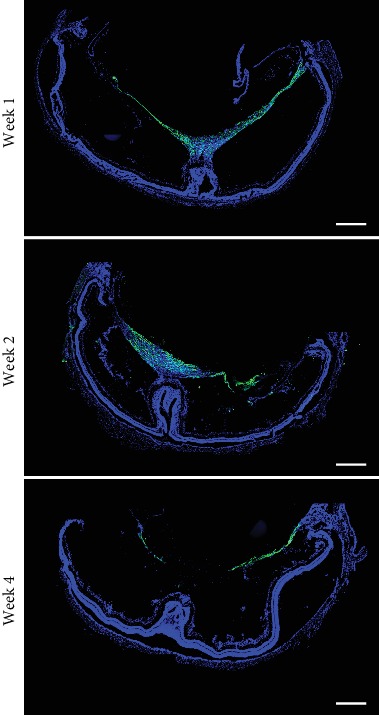
Formation of ERM-like structures by grafted GFP-labeled ASCs in the vitreous chamber at different time points (scale bar = 500 *μ*m). ERM: epiretinal membrane.

**Figure 5:**
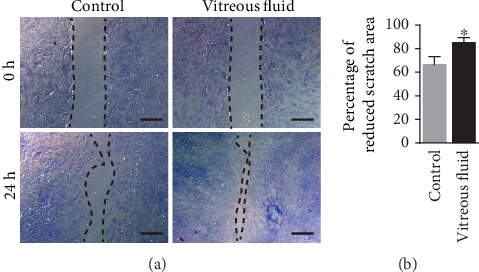
In vitro scratch assay of rASC migration. (a) Representative micrographs of rASC migration. (b) The rate of rASC migration was quantified as percentage of reduced scratch area (*n* = 5). Results are expressed as mean ± SEM; ^∗^*P* < 0.05 compared with the control group.

Previous studies have shown that ERM formed in patients received intravitreal transplantation of bone marrow-derived stem cells and ASCs [17, 19]. We next investigated if ASCs in the vitreous chamber trigger and/or participate in ERM formation, and we observed the distribution of GFP-labeled rASCs in the vitreous chamber. As shown in Figure 4, rASCs migrated from the injection site to the ciliary body, and they formed a membrane (ERM-like structure) and covered the entire retina in the vitreous chamber at week 1 posttransplantation. In addition, rASCs maintained the membrane structure during the entire observation period. rASCs formed an ERM-like structure that was connected to the folded part of the neural retina at the injection site in the vitreous chamber. In order to confirm if vitreous fluid was able to promote rASC to migrate and form a membrane, we further performed an in vitro rASC migration assay and found that the vitreous fluid significantly promoted rASC migration (Figure 5). However, when rASCs were grafted into the subretinal space, cells were aggregated and distributed in a part of the subretinal space. No membrane was formed in the vitreous chamber (Figure 3(c)). Therefore, these results suggested that ASCs easily form an ERM-like structure in the vitreous chamber and induce a fold in the neural retina, subsequently resulting in decreased electric response.

### 3.4. rASC-Formed ERM-Like Structure Contains Several Types of Retinal Cells

**Figure 6:**
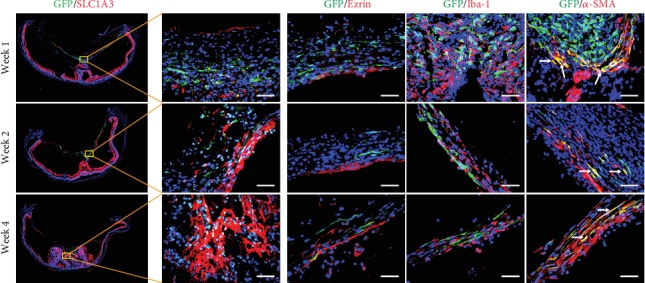
Representative immunofluorescence images of ERM-like structures by grafted GFP-labeled ASCs in the vitreous chamber from different time points. The cellular compartments of ERM-like structures include Müller cells (SLC1A3+), RPE cells (Ezrin+), microglial cells (Iba-1+), and myofibroblast cells (*α*-SMA+); arrows point to the GFP+/*α*-SMA+ cells (scale bar = 50 *μ*m). ERM: epiretinal membrane; RPE cells: retinal pigment epithelial cells.

The cellular components of the ERM include RPE cells, glial cells, and myofibroblasts, and these cells proliferate and migrate onto the surface of the retina and form the ERM. To identify the cellular components of the rASC-formed ERM-like structure in the vitreous chamber, we performed immunostaining. SLC1A3+ Müller cells were in the ERM, and they showed a punctual distribution along the rASC-formed membrane at week 1 posttransplantation and gradually formed a continuous layer attached to the ASC-formed membrane within 4 weeks (Figure 6). Ezrin+ RPE cells were also attached to the rASC-formed membrane, and most of them were distributed between the rASC-formed membrane and retina (Figure 6). Iba-1+ microglial cells are thought to be associated with inflammation [33]. Many microglial cells quickly infiltrated into the rASC-formed membrane at week 1 posttransplantation and stayed in the membrane during the entire observation period (Figure 6). rASCs did not express SLC1A3, Ezrin, and Iba-1 in vitro (Supplementary figure (available here)), which confirmed that SLC1A3+ Müller, Ezrin+ RPE cells, and Iba-1+ microglial cells in the ERM-like membrane were derived from the retina. *α*-SMA+ myofibroblasts are thought to be the main cellular component that results in the contractile ERM [34], subsequently leading to retina folding or detachment. As shown in Figure 6, ASCs differentiated into myofibroblasts, and several GFP-labeled rASCs coexpressed *α*-SMA, which was further confirmed by in vitro immunostaining that part of rASCs expressed *α*-SMA (Supplementary figure). Dedifferentiated RPE cells and Müller cells in the ERM also expressed *α*-SMA. The GFP-negative *α*-SMA+ cells may have been derived from Müller cells and/or RPE cells (Figure 6). These results suggested that rASC-formed ERM-like structures contain Müller cells, RPE cells, and microglial cells and that all of these cells together form an ERM-like structure in the retina.

**Figure 7:**
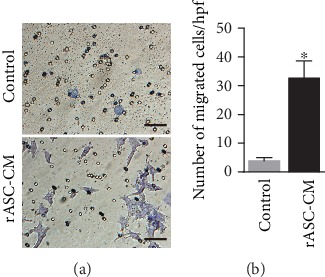
Müller cell migration in transwell. (a) Representative micrographs of Müller cell migration (scale bar = 100 *μ*m). (b) Data are quantified as the number of migrated Müller cells per field (*n* = 9). Results are expressed as mean ± SEM; ^∗^*P* < 0.05 compared with the control group. rASC-CM: rASC-conditioned medium.

ASCs can produce dozens of cytokines and growth factors that affect cell migration [35]. To investigate if ASCs promote retinal cell migration and subsequently enhance ERM-like structure formation, we performed an in vitro migration experiment in a transwell culture plate. ASC-conditioned medium significantly promoted Müller cell migration (Figure 7).

## 4. Discussion

Previous clinical trials have shown that ASC transplantation into the vitreous chamber results in dense vitreous hemorrhage in the eyes, retinal detachment with severe PVR in all patients, and secondary ERM formation in some patients [16, 17]. In addition, transplantation of CD34+ stem cells from the bone marrow into the vitreous chamber also results in ERM formation [19]. However, whether intravitreally transplanted stem cells directly initiate ERM formation or worsen the preexisting ERM remains unknown. In the present study, we did not observe ERM formation in SI-induced RDD rats in neither the PBS groups nor in the rASC subretinal space transplantation groups. rASC vitreous chamber transplantation led to ERM-like structure formation. rASCs formed a membrane as a scaffold in the vitreous chamber, which resulted in a retina fold at the injection site, and retinal cell migration along the scaffold resulted in the formation of the ERM-like structure.

Vitreous fluid contains many kinds of cytokines and growth factors, such as tumor necrosis factor-*α*, interleukin-6, interleukin-8, basic fibroblast growth factor (bFGF), VEGF, platelet-derived growth factor (PDGF), and hepatocyte growth factor (HGF) [36, 37]. The levels of these factors are elevated in vitreous fluid in patients with retinal detachment and are thought to contribute to the onset of PVR [37, 38]. In vitro migration analysis demonstrated that PDGF, HGF, and bFGF, as chemotactic stimuli, promote ASC migration [39]. We also confirmed that vitreous fluid promotes rASC migration in vitro in this study. Therefore, it is conceivable that vitreous fluid promotes intravitreally transplanted ASC migration and formation of a membrane. In addition, the vitreous body contains an extracellular matrix, including collagen [40], which has been confirmed to promote ASC migration [39]. Thus, the vitreous body may provide a scaffold for ASC migration in vivo and promote ERM-like structure formation.

There are two types of ERM, namely, idiopathic and secondary types [21, 22]. The etiologies of idiopathic type involve glial cell migration from ruptured internal limiting membrane, proliferation, and spreading across the retinal surface. The secondary ERM is the complication of eye diseases, such as retinal detachment, diabetic retinopathy, and uveitis. In addition to glial cells, RPE cells migrate from a retinal tear and proliferate, thereby contributing to membrane formation [24, 25]. ERM attaches to the retina, and it contracts and distorts the retina [26]. In this study, ERM formation was not observed in the PBS groups and rASC subretinal space transplantation groups. However, intravitreal transplantation of rASCs induced formation of an ERM-like structure, which indicated that ASCs may be the causative factor of ERM-like structure formation after transplantation into the vitreous chamber. The possible mechanism for ERM-like structure formation may involve ASCs attaching onto the retina at the injection site, thus inducing retina folding and distortion. Simultaneously, ASCs spread along the vitreous body and form a membrane as a scaffold. Retina cells, such as Müller cells, RPE cells, and microglial cells, migrate from the distorted retina and spread along or infiltrate the ASC membrane, forming an ERM-like structure. Thus, formation of an ERM-like structure by ASCs and retinal cells should account for the rapid decrease in ERG electric response.

Abnormal proliferation and migration of retinal glial cells is the critical causative factor for the formation of ERM [41]. Glial cells pass through the inner limiting membrane and migrate onto the retinal surface. The chemokines and growth factors in the vitreous chamber are critical for attracting glial cell migration [42]. It has been reported that HGF stimulates Müller cell migration in vitro [43]. We observed that Müller cells and microglial cells appeared in the vitreous chamber and migrated along or infiltrated into the rASC-formed membrane, subsequently forming an ERM-like structure. ASCs can secrete many growth factors and chemokines, such as HGF, PDGF, VEGF, and bFGF, and these factors directly participate in ERM formation [42]. It is reasonable that these factors form a vitreoretinal gradient to promote glial cell migration. In the present study, rASC-conditioned medium significantly promoted Müller cell migration in a transwell plate. The ciliary body is thought to contain retinal stem cells [44]. Transplanted rASCs formed a membrane in the vitreous cavity and attached to the ciliary body. Retinal stem cells may migrate along the rASC-formed membrane in the vitreous chamber and differentiate into glial cells and RPE cells in the ERM-like structure.

Myofibroblasts produce an extracellular matrix, and they are responsible for ERM contraction [34]. Müller cells in the ERM undergo phenotype change, become myofibroblasts, and express *α*-SMA, which is essential for extracellular matrix contraction, and TGF-*β* promotes this process [45]. The other critical origin of myofibroblasts is thought to be RPE cells. TGF-*β* triggers epithelial-mesenchymal transition of RPE cells and upregulates *α*-SMA expression [46]. Indeed, ASCs can also transform into myofibroblasts [47]. We observed that part of the GFP-labeled ASCs expressed *α*-SMA. However, GFP-negative *α*-SMA+ myofibroblasts may derived from Müller cells or RPE cells. In addition, ASCs may produce TGF-*β*, thus promoting Müller cells and RPE cells to transform into myofibroblasts.

Several factors, including cell quality, immune surveillance, and safety, should be considered before the stem cell-based therapy moves from bench to clinic. Previous studies have reported that intravitreal administration of autologous blood cells resulted in a ERM formation in the vitreous cavity of albino rabbits [18]; AMD or RP patients who received intravitreal injection of bone marrow-derived stem cells developed ERM [19]. Even injection of carbon particles into the vitreous led to ERM formation in rabbits [48]; and we also observed that when human umbilical cord mesenchymal stem cells were transplanted into the subretinal space, if the cells were leaked into the vitreous cavity, ERM was developed (data not shown). These reports together with this study further suggest that the transplantation site is another important factor which may result in different therapeutic outcomes for stem cell-based therapy in patients; at least, the subretinal space rather than the vitreous chamber is the proper transplantation site for the stem cell-based therapy of RDD.

## 5. Conclusions

In the present study, rASCs were autografted into the vitreous chamber of rats, and vitreous fluid promoted rASC migration to form a membrane. In addition, a retinal fold formed at the rASC injection site, and the rASC-formed membrane acted as a scaffold to promote glial cell and RPE cell migration, which formed an ERM-like structure composed of rASCs and retinal cells. Although intravitreal transplantation of rASCs reduced the formation of rosettes in the retina and reduced the apoptosis of neural retinal cells, the ERM-like structure and retina fold formation may be the main reasons leading to the extinguished electric response of the eye. Considering that subretinal space transplantation of rASCs recovered the electric response to a certain degree, we concluded that the transplantation site affects the ASC-based therapeutic outcomes and the vitreous chamber is not a good transplantation site for ASC-based therapy for RDD. Thus, deliberate decisions should be made before transplantation of stem cells into the vitreous chamber to treat RDD in clinical trials.

## Data Availability

The data used to support the findings of this study are available and included within the article and the supplementary information file.

## Abbreviations

AMD: Age-related macular degeneration
AKP: Alkaline phosphatase
ARVO: The Association for Research in Vision and Ophthalmology
bFGF: Basic fibroblast growth factor
BSA: Bovine serum albumin
ERG: Electroretinogram
ERM: Epiretinal membrane
GCL: Ganglion cell layer
GFP: Green fluorescent protein
HGF: Hepatocyte growth factor
INL: Inner nuclear layer
ONL: Outer nuclear layer
PBS: Phosphate-buffered saline
PDGF: Platelet-derived growth factor
PFA: Paraformaldehyde
PRs: Photoreceptors
PVR: Proliferative vitreoretinopathy
rASCs: Rat adipose-derived stem cells
RDD: Retinal degenerative disease
RP: Retinitis pigmentosa
RPE: Retinal pigment epithelial
SD: Sprague-Dawley
SI: Sodium iodate
TGF*β*1: Transforming growth factor *β*1
TUNEL: Terminal deoxynucleotidyl transferase-mediated deoxyuridine triphosphate nick end labeling
VEGF: Vascular endothelial growth factor.

## Supplementary Materials

Supplementary Materials Immunostaining of rASCs. The rASCs were grown to 80% confluence in a 24-well culture plate and fixed with 4% PFA, permeabilized with 0.25% Triton X-100 for 2 min, washed with PBS, and then blocked with 2% BSA in PBS. Then, the cells were separately incubated with the primary antibodies against SLC1A3, Ezrin, Iba-1, and *α*-SMA, each diluted 1 : 500 overnight at 4°C. They were then washed three times with PBS, followed by incubation with the fluorescent secondary antibodies (1 : 1000) overnight. DAPI was used to indicate the nucleus. The cells were examined under a fluorescence microscope (Olympus IX73, Tokyo, Japan).
