# Supplementary material for: Transplantation Site Affects the Outcomes of Adipose-Derived Stem Cell-Based Therapy for Retinal Degeneration
Source: Stem Cells Int. 2020 Jan 6;2020:9625798. doi: 10.1155/2020/9625798 (PMC7199575; doi:10.1155/2020/9625798)
Supplement: Supplementary Materials — Immunostaining of rASCs. The rASCs were grown to 80% confluence in a 24-well culture plate and fixed with 4% PFA, permeabilized with 0.25% Triton X-100 for 2 min, washed with PBS, and then blocked with 2% BSA in PBS. Then, the cells were separately incubated with the primary antibodies against SLC1A3, Ezrin, Iba-1, and α-SMA, each diluted 1 : 500 overnight at 4°C. They were then washed three times with PBS, followed by incubation with the fluorescent secondary antibodies (1 : 1000) overnight. DAPI was used to indicate the nucleus. The cells were examined under a fluorescence microscope (Olympus IX73, Tokyo, Japan). [file 9625798.f1.pdf]

Supplementary Materials

The rASCs were grown to 80% confluence in 24-well culture palte and fixed with 4% PFA, permeabilized with 0.25% Triton X-100for 2 min, washed with PBS, and then blocked with 2% BSA in PBS. Then the cells were separately incubated with the primary antibodies against SLC1A3, Ezrin, Iba-1 and α-SMA, each diluted 1:500 over night at 4°C. They were then washed three times with PBS, followed by incubation with the fluorescent secondary antibodies (1:1000) overnight. DAPI was used to indicate the nucleus. The cells were examined under a fluorescence microscope (Olympus IX73, Tokyo, Japan).

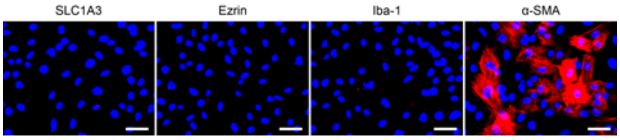

Supplementary figure. Representative images of the expressions of SLC1A3, Ezrin, Iba-1 and α-SMA in the rASCs. SLC1A3, Ezrin and Iba-1 were not expressed in rASCs, and α-SMA was expressed in part of rASCs (scale bar = 50 μm).
